# Establishment of ICU Mortality Risk Prediction Models with Machine Learning Algorithm Using MIMIC-IV Database

**DOI:** 10.3390/diagnostics12051068

**Published:** 2022-04-24

**Authors:** Ke Pang, Liang Li, Wen Ouyang, Xing Liu, Yongzhong Tang

**Affiliations:** 1 Department of Anesthesiology, Third Xiangya Hospital, Central South University, Changsha 410013, China; pangke97@gmail.com (K.P.); yangwenou@126.com (W.O.); xinxingmail@csu.edu.cn (X.L.); 2 Department of Gastrointestinal Surgery, Third Xiangya Hospital, Central South University, Changsha 410013, China; liliang97116@csu.edu.cn

**Keywords:** machine learning, postoperative death, prediction model

## Abstract

**Objective:** The mortality rate of critically ill patients in ICUs is relatively high. In order to evaluate patients’ mortality risk, different scoring systems are used to help clinicians assess prognosis in ICUs, such as the Acute Physiology and Chronic Health Evaluation III (APACHE III) and the Logistic Organ Dysfunction Score (LODS). In this research, we aimed to establish and compare multiple machine learning models with physiology subscores of APACHE III—namely, the Acute Physiology Score III (APS III)—and LODS scoring systems in order to obtain better performance for ICU mortality prediction. **Methods:** A total number of 67,748 patients from the Medical Information Database for Intensive Care (MIMIC-IV) were enrolled, including 7055 deceased patients, and the same number of surviving patients were selected by the random downsampling technique, for a total of 14,110 patients included in the study. The enrolled patients were randomly divided into a training dataset (n = 9877) and a validation dataset (n = 4233). Fivefold cross-validation and grid search procedures were used to find and evaluate the best hyperparameters in different machine learning models. Taking the subscores of LODS and the physiology subscores that are part of the APACHE III scoring systems as input variables, four machine learning methods of XGBoost, logistic regression, support vector machine, and decision tree were used to establish ICU mortality prediction models, with AUCs as metrics. AUCs, specificity, sensitivity, positive predictive value, negative predictive value, and calibration curves were used to find the best model. **Results:** For the prediction of mortality risk in ICU patients, the AUC of the XGBoost model was 0.918 (95%CI, 0.915–0.922), and the AUCs of logistic regression, SVM, and decision tree were 0.872 (95%CI, 0.867–0.877), 0.872 (95%CI, 0.867–0.877), and 0.852 (95%CI, 0.847–0.857), respectively. The calibration curves of logistic regression and support vector machine performed better than the other two models in the ranges 0–40% and 70%–100%, respectively, while XGBoost performed better in the range of 40–70%. **Conclusions:** The mortality risk of ICU patients can be better predicted by the characteristics of the Acute Physiology Score III and the Logistic Organ Dysfunction Score with XGBoost in terms of ROC curve, sensitivity, and specificity. The XGBoost model could assist clinicians in judging in-hospital outcome of critically ill patients, especially in patients with a more uncertain survival outcome.

## 1. Introduction

As the number of critically ill patients is increasing, the demand for intensive care units (ICUs) has also substantially increased. Increasing demand for critical care has made capacity limitations commonplace in ICUs [1]. Critically ill patients admitted to ICUs are at a high risk of mortality [2]. Previous studies have indicated that the overall mortality rate was 20.5–43% among patients with an ICU stay, and the most common causes of death among patients in ICUs were sepsis, cardiac arrest, pneumonia, and cardiac arrhythmia [3]. Previous evidence has suggested that the severity and extent of disease upon admission to the ICU are strongly associated with ICU in-hospital mortality [4]. Therefore, the outcome of ICU patients predicted by multifactorial scores upon admission to the ICU is critical for long-term treatment and humanistic care [5]. At present, when patients are admitted to the ICU, they are scored with scales such as the Acute Physiology and Chronic Health Evaluation III (APACHE III) score, the Logistic Organ Dysfunction Score (LODS), and the Sequential Organ Failure Assessment (SOFA) [6,7]. Some scales, including SOFA, Systemic Inflammatory Response Syndrome (SIRS), and APACHE II, have been used to predict outcomes in critically ill patients and achieved adequate results [8,9].

Machine learning techniques have been widely used in clinics, ranging from diagnosis to predicting survival outcomes [10,11]. For ICU mortality prediction, the current prognosis models employ the logistic regression classifier or the single long short-term memory (LSTM) classifier [12] and single scoring system [13]. However, logistic regression constructs linear decision boundaries, and therefore, nonlinear problems may have relatively poor prediction results with logistic regression [14]. Previous research showed that an ensemble machine learning algorithm could have better prediction performance with Simplified Acute Physiology Score (SAPSII) and SOFA scores as input variables compared with logistic regression [15]. The XGBoost algorithm has been used to predict mortality based on the MIMIC-III database. A study used admission and laboratory variables to construct an XGBoost model to predict in-hospital mortality among patients with heart failure and achieved a high AUC of 0.84 [16]. Another study used the XGBoost algorithm to predict all-cause mortality based on the MIMIC-III database with some acute physiology variables and chronic conditions and achieved the highest AUC of 0.86 compared with other models [17].

It remains to be seen if we can achieve higher accuracy of survival outcome prediction by taking each score of both APS III and LODS scoring systems as the input features of nonlinear classifiers based on an ensemble machine learning algorithm. There were a few studies that used APS II or LODS to predict mortality in the ICU. A study on assessing the physiological instability of pediatric intensive care unit patients found that APS III could be sensitive to small changes in physiological status [18]. A previous study based on the MIMIC-III database used APS III data as input variables to construct a model to predict mortality among trauma patients with acute respiratory distress syndrome and found that the model achieved an AUC of 0.718 [19]. Another study used LODS to predict all-cause 30-day mortality and achieved an AUC of 0.733 among intensive care patients with sepsis based on the MIMIC-III database. As a result, we chose two kinds of scoring systems to construct models and achieve higher prediction performance [20]. There are few other research works that combine two scoring systems to predict mortality in ICUs.

We aimed to integrate the physiology subscores of APACHE III—namely, the APS III scoring system—and the LODS scoring system, and compare four different machine learning models (XGBoost [21], logistic regression, SVM, and decision tree) based on the data of 14,110 patients in the MIMIC-IV database [22] to predict the different performances of ICU patient mortality.

## 2. Methods

### 2.1. Data Source and Population

The study data were taken from the Medical Information Mart for Intensive Care (MIMIC)-IV database [22]. MIMIC-IV is a large, single-center database with more than 70,000 patients. For this study, we selected 67,748 adult patients with LODS scores and acute physiology subscores as part of APACHE III scores in the MIMIC-IV database and performed a retrospective review.

**Figure 1:**
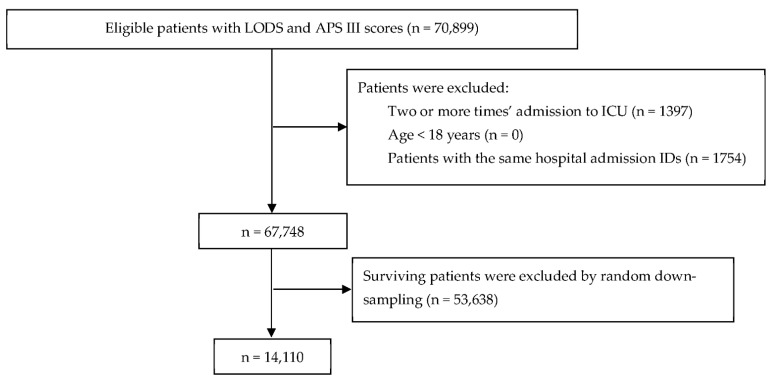
Flow chart.

The inclusion criteria were patients admitted to the ICU for the first time who were older than 18 years. The first ICU admission was considered when a subject had multiple admissions to the ICU. The exclusion criteria were patients with admission to an ICU two or more times, patients younger than 18 years, and patients with the same hospital admission IDs. We did not exclude patients with any diseases, similar to the method used in previous studies [23]. Class imbalance is a major problem in ICU datasets, as the number of deceased patients (7055, 10.4%) is much lower than the number of living patients (60,693, 89.6%). Methods for dealing with datasets with class imbalance include resampling [24,25] and classifying cost functions [26]. Downsampling is a kind of resampling that entails decreasing the number of records in the majority class with more samples. We used random downsampling to randomly select the same number of positive samples as the negative samples from the original dataset of 60,693 patients [27]. After random downsampling, a total of 14,110 patients (7055 in-hospital deceased patients and 7055 surviving patients) were considered in the study. The sample size was sufficiently large, and no sample size calculation was undertaken. The flow chart of the study is shown in Figure 1. PostgreSQL was used to extract clinical information, including age, sex, weight, admission type, Logistic Organ Dysfunction Score (LODS), and Acute Physiology Score III (APS III) on the PostgreSQL database server (version 10).

### 2.2. Selection of Variables

The LODS score is based on six different scores, one each for the respiratory, cardiovascular, hepatic, coagulation, renal, and neurological systems. APS III scores include heart rate score, mean blood pressure score, temperature score, respiratory rate score, PaO_2_-aadO_2_ score, hematocrit score, white blood count score, serum creatinine score, urine output score, blood urea nitrogen score, sodium score, albumin score, bilirubin score, glucose score, acid base score, Glasgow Coma Scale score, and total APS III score.

### 2.3. Data Analysis and Model Construction

After employing the random downsampling technique to select surviving patients, the dataset was partitioned into the training set (70%) and the testing set (30%). After the completion of the feature engineering, the machine learning algorithms, including XGBoost, support vector machine (SVM), logistic regression (LR), and decision tree, were used to construct the models [28]. Receiver operating characteristic (ROC) curve analysis was considered as a metric to tune model parameters. Grid search and 5-fold cross-validation [29] were performed for hyperparameter optimization and the construction of prediction models. The AUCs, sensitivity, specificity, positive predictive rate, and negative predictive rate were calculated, and calibration curves [30] were plotted to evaluate the advantages or disadvantages of the models.

We performed statistical analyses using the *sklearn* machine learning package (0.24.2), xgboost package (1.5.0), and shap package (0.40.0) in Python 3.7.4 and R 4.1.0 programs. The normality of continuous variables was analyzed by the normality test. Continuous variables with normal distribution were expressed as mean ± standard deviations and continuous variables with non-normal distribution were expressed as median [IQR]. Categorical data are shown as numbers (percent). Group comparisons for continuous data with normal distribution were calculated with Student’s t-test, while continuous data with non-normal distribution were calculated with the Kruskal–Wallis test, and categorical data were compared using χ^2^ or Fisher’s exact test with the tableone package in R 4.1.0. Effects with *p*-values smaller than 0.05 were considered significant.

## 3. Results

The pre- and post-sampling characteristics of the study subjects are presented in Table 1. The data show significant differences between surviving and in-hospital deceased patients in terms of admission type, weight, neurological score, cardiovascular score, renal score, pulmonary score, hematological score, hepatic score, total LODS score in the LODS scoring system, heart rate score, mean blood pressure score, temperature score, PaO2-aadO2 score, white blood count score, serum creatinine score, urine output score, blood urea nitrogen score, blood sodium score, albumin score, bilirubin score, glucose score, acid base score, Glasgow Coma Scale score, and total APS III score in the APS III scoring system (*p* < 0.001). However, there were no statistical differences between surviving and in-hospital deceased patients in respiratory rate score, hematocrit score, and gender.

**Figure 2:**
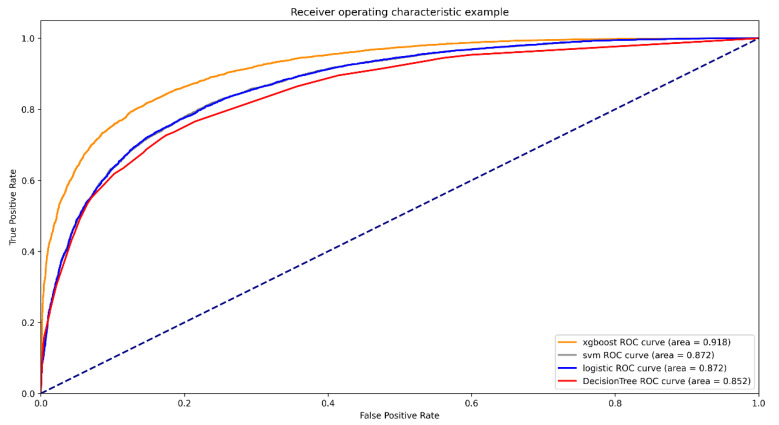
ROCs of different models.

**Figure 3:**
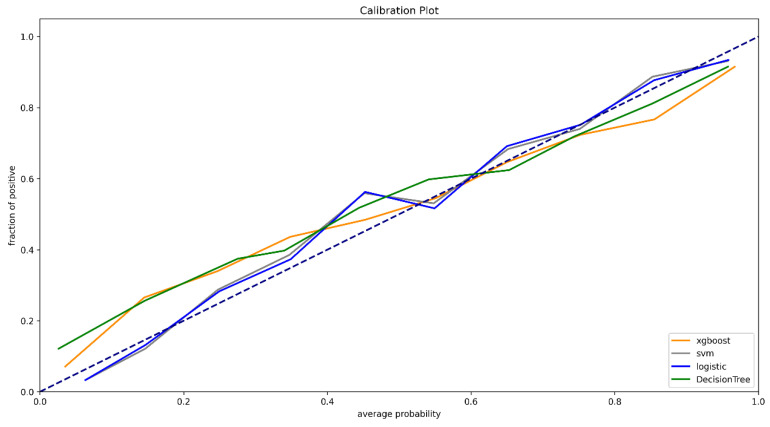
Calibration curve.

**Figure 4:**
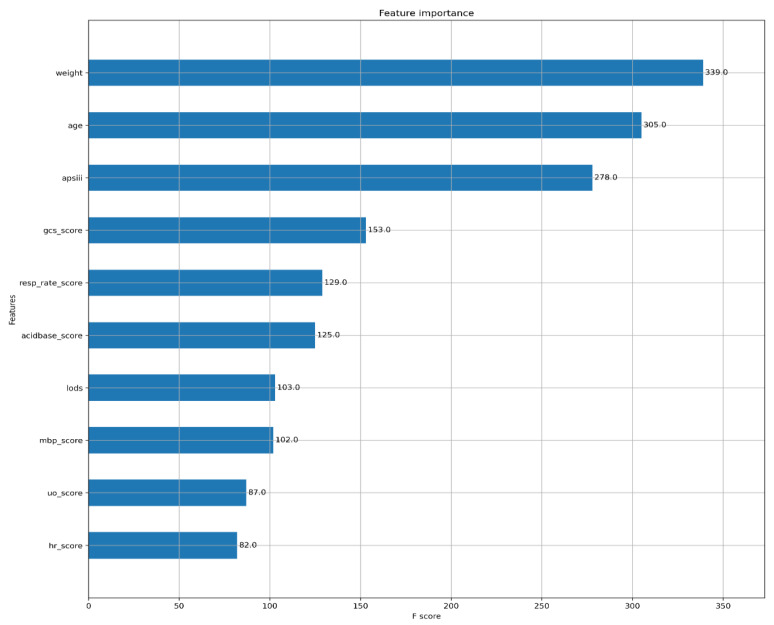
Feature importance plot of XGBoost.

For the prediction of mortality in ICU patients (Figure 2), the AUC of the XGBoost model was 0.918 (95%CI, 0.915–0.922). The AUCs of logistic regression, SVM, and decision tree were 0.872 (95%CI, 0.867–0.877), 0.872 (95%CI, 0.867–0.877), and 0.852 (95%CI, 0.847–0.857), respectively (Table 2). XGBoost showed better accuracy, sensitivity, specialty, positive predictive value, and negative predictive value compared with SVM, logistic regression, and decision tree. The calibration curves of logistic regression and SVM performed better than the other two models in the low and high probability range (0–40% and 70–100%), while the calibration curve of XGBoost performed better in the medium probability range of 40–70% (Figure 3). The XGBoost feature importance plot shows that apart from total LODS score, total APS III score, weight, and age, the three most important characteristics in predicting ICU mortality were Glasgow Coma Scale score, respiratory rate score, and acid base score (Figure 4). The SHAP bee swarm plot shows the SHAP value importance of all features in the XGBoost model (Supplementary Figure S2), and the results show that in the plot, the Glasgow Coma Scale score, acid base score, and urine output score were the three most important features in predicting mortality [31]. The hyperparameters of the models are shown in Supplementary Table S1.

## 4. Discussion

Critical illness in the ICU is associated with in-hospital mortality and substantial economic burden. The in-hospital mortality in ICUs accounts for 20–50% of all in-hospital deaths [32,33], and the ICU accounts for 22% of the aggregate costs [34] for all hospitalizations, or nearly USD 81.3 billion in 2005 [35]. Early aggressive therapy can retard progression and control disease. However, it is difficult for clinicians to predict which patients will worsen and to evaluate the risk of not treating patients or if they will respond to specific therapy. As a result, better prediction models are needed to predict the mortality risk of critically ill patients in the ICU. Several prognostic scoring systems in ICUs have been developed to predict the outcome of patients. The advantages of such scoring systems are that they are easy to measure and interpret and are less prone to measurement and calculation errors. In this study, we used two prognostic scoring systems (LODS and APS III, the physiology subscore part of the APACHE III scoring system) as input variables, as more variables could provide better prediction performance [36,37]. The Logistic Organ Dysfunction Score (LODS) system is a common and important scoring system. LODS scores are used to assess six organ or system states and record the worst score within 24 h after admission to the hospital. The organ scoring system assesses for dysfunction of neurological, cardiovascular, renal, pulmonary, hematological, and hepatic systems [38]. As a weighted system, LODS is summed by six subscores, ranging from 0 to 5, and each subscore represents an organ or system’s function or state. However, for the respiratory and hematological systems, the highest score is 3 points, and for the hepatic system, the highest is 1 point. Since its development in 1996 [39], it has been widely used for assessing mortality in ICUs. The Acute Physiology and Chronic Health Evaluation system was introduced in the early 1980s and has experienced three major revisions [40]. Although the APACHE II model is old, and new scoring systems have been developed using more recent cohorts and better features, APACHE II is still widely used in clinical practice [41]. The APACHE III scoring system was developed in 1991. Compared to the APACHE II scoring system, APACHE III performs better in terms of correct classification and the AUCs [42]. The APACHE III scores several factors, including clinical complications, vital signs, and partial blood biochemical examination results [43]. A higher score of LODS or APACHE III is associated with high mortality in the ICU. Although some studies took APACHE III as features to establish machine learning models, there is little literature on using LODS or APS III data as partial input variables at present.

Our study aimed to compare the predictive power mortality between four different machine learning models using subscores of LODS and APS III in predicting in-hospital mortality of ICU patients. In the dataset, the mortality rate of ICU patients was 10.4%. Of the four models, XGBoost showed the best performance in predicting mortality, followed by SVM, logistic regression, and decision tree. Moreover, calibration curves were plotted to evaluate the clinical usefulness of different mortality ranges. The results showed that in the uncertain medium mortality risk range (40–70%), XGBoost was more valuable than logistic regression and SVM models.

As the most widely used model, logistic regression has been used to diagnose diseases and predict outcomes. A study based on a Spanish ICU database revealed that a logistic regression model could achieve an AUC of 0.82 with APACHE III data as input variables, which showed prediction ability to some extent [36]. Another study based on an American ICU database found that using APACHE IV data as input variables could achieve high prediction results [44]. Previous studies found that logistic regression and artificial neural network (ANN) had similar performance when the sample size was adequate [45]. Although logistic regression could not provide a nonlinear decision boundary, it still achieved suitable prediction results. However, more studies revealed that, compared with logistic regression, ANN demonstrated a better degree of discrimination in complex clinical situations [46]. And another research revealed that using ANN to predict early hospital mortality in acute pancreatitis in MIMIC-III could achieve higher prediction performance compared with logistic regression [47]. This may be because ANNs have an inherently flexible nature that suits more complicated interactions between the clinical input variables. In comparison, logistic regression lacks modeling for complex interactions in clinical issues. Some studies found that logistic regression had a relatively worse performance in AUCs, prediction accuracy, or other metrics [36]. Meanwhile, there is research revealing a better discrimination in predicting ICU mortality using XGBoost and gradient-boosted decision trees (GBDT) models compared to SVM [48]. However, a better performance using SVM classification to predict mortality risk for ICU patients with sepsis compared with logistic regression has also been shown [49]. That might be because, depending on the particular dataset or subject population, nonlinear classifiers (XGBoost and SVM) could obtain better predictive performance compared with linear classifiers (logistic regression), which means researchers need to take practical issues into account and select the optimal model.

Some previous studies used vital signs and laboratory variables available in conventional clinical scoring systems as input features to predict mortality based on the MIMIC-III, MIMIC-IV, and eICU databases with recurrent neural networks and achieved similar prediction performance [31]. Another study used partial vital signs and Glasgow Coma Scale scores at different time points after admission to the hospital as input features to predict mortality based on the MIMIC-III database with a convolutional neural network-based prediction model for multivariate time series [50]. The above studies used SHAP or heatmaps to interpret the importance or contribution of the models. However, the studies used a single scoring system as input variables. In contrast, our study selected all subscores of LODS and APS III scoring systems as input variables, as they were completed within the first 24 h of admission to ICU [51], and we used SHAP to explore the features’ importance following the method employed in previous studies. Additionally, we used the calibration curve to find the best prediction range of different models. Previous studies showed that constructing models based on SVM, neural network, and logistic regression with SOFA scores as input variables to predict ICU mortality all performed well [23]. A study using APACHE III as variables to construct an XGBoost model based on the MIMIC-III database showed that XGBoost could perform better in accuracy, sensitivity, specificity, and AUC [52], and the comparisons between XGBoost and other models (including logistic regression and multilayer perceptron models) were statistically significant. Our research drew a similar conclusion, as XGBoost had advantages in accuracy, AUCs, and discrimination ability compared with SVM, logistic regression, and decision tree. However, among the population with high mortality probability (more than 70%) and low mortality probability (less than 40%), the calibration of SVM and logistic regression was better than XGBoost, while among the population with medium mortality probability (40–70%), XGBoost had advantages in calibration and discrimination compared with SVM and logistic regression. As a result, in terms of ROC curve, sensitivity, and specificity, for patients whose prognosis is difficult to predict by clinical experience, XGBoost performs better.

Although the importance of variables in XGBoost is shown in Figure 4, the recognition of variables’ importance and mortality in the ICU could not be completely explained. However, the reason why the variables of weight, age, and APS III total score had high importance was because the values of the three variables were relatively large compared with other scores. The three variables of the Glasgow Coma Scale score, respiratory rate score, and acid base score were the most important variables. The SHAP bee swarm plot shown in Supplementary Figure S2 showed a similar result, that the Glasgow Coma Scale score, respiratory rate score, and acid base score were the three most important variables. As a result, special attention should be paid to these physiological indices. This result is consistent with previous studies. A study by Daniel found that the Glasgow Coma Scale score dominates in predicting 30-day mortality in a mixed ICU with admission Sequential Organ Failure Assessment scores as input variables [53]. Another study revealed that the Glasgow Coma Scale was more suitable for early in-hospital death assessment among patients with acute head injury [54]. A study by Piotr found that in multivariate analysis, the Glasgow Coma Scale score was the most important variable in critically ill surgical and nonsurgical patients [55]. There are few studies about respiratory rate predicting value for mortality. A multicenter study developed a machine learning analysis with age, heart rate, and respiratory rate as input features and found that the two most important prediction factors were respiratory rate and heart rate [56]. Considering acid base, a study by Anja found that in the ICU, some acid base imbalance factors (including lactate, base excess, and pH) were all suitable predictors of mortality [57].

Compared with previous related studies, our study introduced each score in the APS III and LODS scales to predict mortality in the ICU based on a newly released database and achieve better prediction performance and used calibration curves to judge the best prediction range of different patients with different mortality risk. In the SHAP plots of value importance and feature importance of XGBoost, we explained the most influential physiological conditions for survival. Clinicians can judge patients’ mortality probability by whether the patients were at high or low mortality risk.

The strengths of this study rest on several aspects. First, we used the updated MIMIC-IV database with complex and comprehensive information. Second, relatively novel machine learning methods were used to replace the traditional logistic regression, and the results showed better performance of XGBoost methods than the conventional logistic regression model. Third, better statistical methods were used to replace traditional methods, such as 5-fold cross-validation to evaluate the model, and the results showed that XGBoost had better performance. Fourth, we plotted calibration curves and found that patients with different mortality risks could be assessed with different machine learning models. In our study based on the MIMIC-IV database, the relatively certain in-hospital outcome of patients with high or low mortality probability (0–40% and 70–100%) could be predicted with a logistic regression model or SVM, while the relatively uncertain survival outcome of patients with medium mortality probability (40–70%) could be predicted with XGBoost. Fifth, we used the SHAP bee swarm to explain the importance of all input features. Additional different machine learning models should be developed, aiming to predict the outcomes of critically ill patients with different scores.

However, there were also limitations in the present study. First, it was a single-center retrospective study. Thus, further prospective multicenter studies are needed to validate the current results. Second, this observational study used the random downsampling technique to select surviving patients, which might result in some information loss and potential bias. A better sampling technique or more datasets in order to obtain balanced datasets can achieve better performance [58].

## 5. Conclusions

Compared with models with a single scoring system to predict mortality, our models of data analysis provide strong evidence for the accuracy of predicting mortality in the ICU with the APS III–LODS-based scoring system. In conclusion, this study showed that a machine learning method based on XGBoost could perform better than conventional logistic regression and support vector machine models. The Glasgow Coma Scale, acid base score, urine output, and respiratory rate should be considered in order to improve clinical prognosis. The XGBoost model could assist clinicians in judging in-hospital outcome of critically ill patients, especially in patients with a more uncertain survival outcome.

## Figures and Tables

**Table 1:** Baseline data of participants.

Variable (Score)	Dataset before Downsampling			Dataset after Downsampling		
	Survived (60,693)	Dead (7055)	*p*	Survived (7055)	Dead (7055)	*p*
Female ^1^	26,774 (44.1)	3235 (45.9)	0.006	3193 (45.3)	3235 (45.9)	0.488
Age ^3^	64.37 ± 17.10	71.44 ± 15.23	<0.001	64.25 ± 17.32	71.44 ± 15.23	<0.001
Weight ^3^	81.48 ± 26.00	77.34 ± 23.89	<0.001	81.08 ± 26.33	77.34 ± 23.89	<0.001
Emergency ^1^	43,724 (72.0)	6016 (85.3)	<0.001	5102 (72.3)	6016 (85.3)	<0.001
LODS ^2^	3.00 [2.00, 5.00]	8.00 [5.00, 11.00]	<0.001	3.00 [2.00, 6.00]	8.00 [5.00, 11.00]	<0.001
Neurologic ^2^	0.00 [0.00, 1.00]	1.00 [0.00, 3.00]	<0.001	0.00 [0.00, 1.00]	1.00 [0.00, 3.00]	<0.001
Cardiovascular ^2^	0.00 [0.00, 1.00]	1.00 [0.00, 1.00]	<0.001	0.00 [0.00, 1.00]	1.00 [0.00, 1.00]	<0.001
Renal ^2^	1.00 [1.00, 3.00]	3.00 [1.00, 5.00]	<0.001	1.00 [1.00, 3.00]	3.00 [1.00, 5.00]	<0.001
Pulmonary ^2^	0.00 [0.00, 1.00]	1.00 [0.00, 3.00]	<0.001	0.00 [0.00, 1.00]	1.00 [0.00, 3.00]	<0.001
Hematologic ^2^	0.00 [0.00, 0.00]	0.00 [0.00, 0.00]	<0.001	0.00 [0.00, 0.00]	0.00 [0.00, 0.00]	<0.001
Hepatic ^2^	0.00 [0.00, 1.00]	1.00 [0.00, 1.00]	<0.001	0.00 [0.00, 1.00]	1.00 [0.00, 1.00]	<0.001
APS III ^2^	39.00 [29.00, 52.00]	73.00 [53.00, 95.00]	<0.001	39.00 [29.00, 52.00]	73.00 [53.00, 95.00]	<0.001
Heart rate ^2^	1.00 [0.00, 5.00]	5.00 [0.00, 7.00]	<0.001	1.00 [0.00, 5.00]	5.00 [0.00, 7.00]	<0.001
Mean pressure ^2^	9.00 [7.00, 15.00]	15.00 [7.00, 15.00]	<0.001	9.00 [7.00, 15.00]	15.00 [7.00, 15.00]	<0.001
Temperature ^2^	0.00 [0.00, 0.00]	0.00 [0.00, 2.00]	<0.001	0.00 [0.00, 0.00]	0.00 [0.00, 2.00]	<0.001
Respiratory rate ^2^	6.00 [6.00, 8.00]	6.00 [6.00, 8.00]	<0.001	6.00 [6.00, 8.00]	6.00 [6.00, 8.00]	0.001
PaO_2_-aadO_2_^2^	0.00 [0.00, 0.00]	0.00 [0.00, 0.00]	<0.001	0.00 [0.00, 0.00]	0.00 [0.00, 0.00]	<0.001
Hematocrit ^2^	3.00 [3.00, 3.00]	3.00 [3.00, 3.00]	0.670	3.00 [3.00, 3.00]	3.00 [3.00, 3.00]	0.735
White blood count ^2^	0.00 [0.00, 0.00]	0.00 [0.00, 1.00]	<0.001	0.00 [0.00, 0.00]	0.00 [0.00, 1.00]	<0.001
Creatinine ^2^	0.00 [0.00, 3.00]	4.00 [0.00, 7.00]	<0.001	0.00 [0.00, 4.00]	4.00 [0.00, 7.00]	<0.001
Urine output ^2^	4.00 [0.00, 5.00]	5.00 [4.00, 8.00]	<0.001	4.00 [0.00, 5.00]	5.00 [4.00, 8.00]	<0.001
Blood urea nitrogen ^2^	2.00 [0.00, 7.00]	7.00 [7.00, 11.00]	<0.001	2.00 [0.00, 7.00]	7.00 [7.00, 11.00]	<0.001
Blood sodium ^2^	0.00 [0.00, 0.00]	0.00 [0.00, 2.00]	<0.001	0.00 [0.00, 0.00]	0.00 [0.00, 2.00]	<0.001
Albumin ^2^	0.00 [0.00, 0.00]	0.00 [0.00, 0.00]	<0.001	0.00 [0.00, 0.00]	0.00 [0.00, 0.00]	<0.001
Bilirubin ^2^	0.00 [0.00, 0.00]	0.00 [0.00, 0.00]	<0.001	0.00 [0.00, 0.00]	0.00 [0.00, 0.00]	<0.001
Glucose ^2^	0.00 [0.00, 3.00]	0.00 [0.00, 3.00]	<0.001	0.00 [0.00, 3.00]	0.00 [0.00, 3.00]	<0.001
Acid base ^2^	0.00 [0.00, 2.00]	3.00 [0.00, 9.00]	<0.001	0.00 [0.00, 2.00]	3.00 [0.00, 9.00]	<0.001
Glasgow Coma Scale ^2^	0.00 [0.00, 3.00]	3.00 [0.00, 29.00]	<0.001	0.00 [0.00, 3.00]	3.00 [0.00, 29.00]	<0.001
Hypertension ^1^	38,236 (63.0)	4608 (65.3)	<0.001	4399 (62.4)	4608 (65.3)	<0.001
Ischemic heart disease ^1^	20,317 (33.5)	2568 (36.4)	<0.001	2307 (32.7)	2568 (36.4)	<0.001
Diabetes ^1^	18,001 (29.7)	2135 (30.3)	0.301	2053 (29.1)	2135 (30.3)	0.136
Chronic pulmonary disease ^1^	15,248 (25.1)	1916 (27.2)	<0.001	1721 (24.4)	1916 (27.2)	<0.001
Cerebrovascular disease ^1^	8919 (14.7)	1630 (23.1)	<0.001	1072 (15.2)	1630 (23.1)	<0.001

Data are number of subjects (percentage) or median [IQR]. ^1^ Chi-square test or Fisher’s exact test was used to compare the percentage between participants between surviving and deceased patients. ^2^ Kruskal–Wallis test was used to compare the median [IQR] between surviving and deceased patients. ^3^ Student’s *t*-test was used to compare the mean ± standard deviations between surviving and deceased patients.

**Table 2:** AUC, accuracy, sensitivity, specialty, positive predictive value, and negative predictive value of different models.

Models	ROC (95%CI)	Accuracy	SEN	SPE	PPV	NPV
XGBOOST	0.918 (0.915–0.922)	0.834	0.822	0.846	0.842	0.826
SVM	0.872 (0.867–0.877)	0.789	0.773	0.805	0.799	0.780
Logistic regression	0.872 (0.867–0.877)	0.787	0.756	0.818	0.806	0.771
Decision Tree	0.852 (0.847–0.857)	0.776	0.727	0.825	0.806	0.752

## Data Availability

Restrictions apply to the availability of these data. Data were obtained from MIMIC-IV and are available at https://physionet.org/content/mimiciv/1.0/ (accessed on 1 October 2021) with the permission of PhysioNet.

## Supplementary Materials

The following supporting information can be downloaded at: https://www.mdpi.com/article/10.3390/diagnostics12051068/s1, Figure S1: ROC curves of training dataset (A) and testing dataset (B); Figure S2: SHAP values importance of all features in the Xgboost model; Table S1: Hyperparameter values of models.
