# Supplementary material for: Establishment of ICU Mortality Risk Prediction Models with Machine Learning Algorithm Using MIMIC-IV Database
Source: Diagnostics (Basel). 2022 Apr 24;12(5):1068. doi: 10.3390/diagnostics12051068 (PMC9139972; doi:10.3390/diagnostics12051068)
Supplement: Supplementary file 1 [file diagnostics-12-01068-s001.zip › diagnostics-1653214-supplementary.pdf]

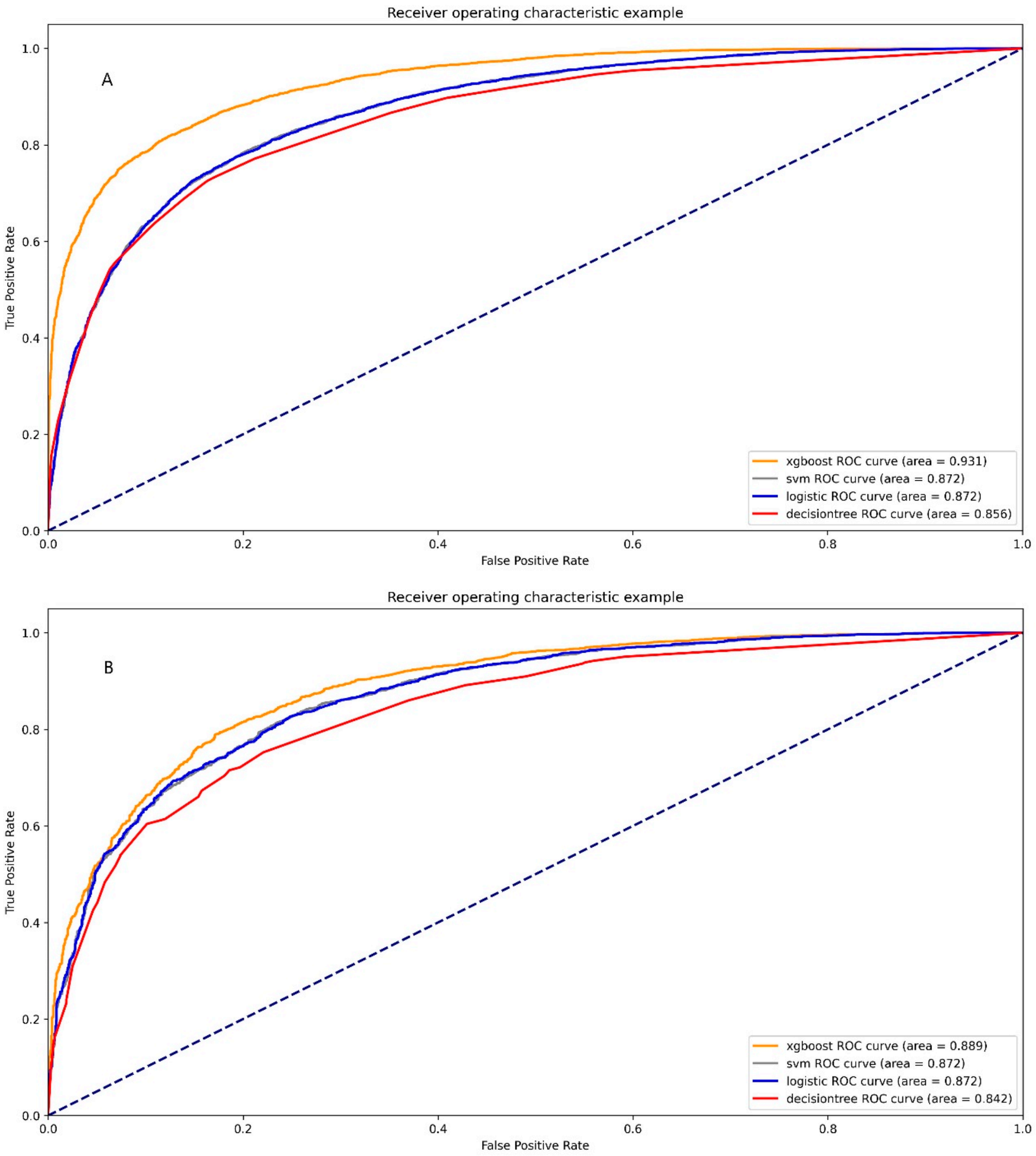

Supplementary Figure S1 ROC curves of training dataset (A) and testing dataset (B)

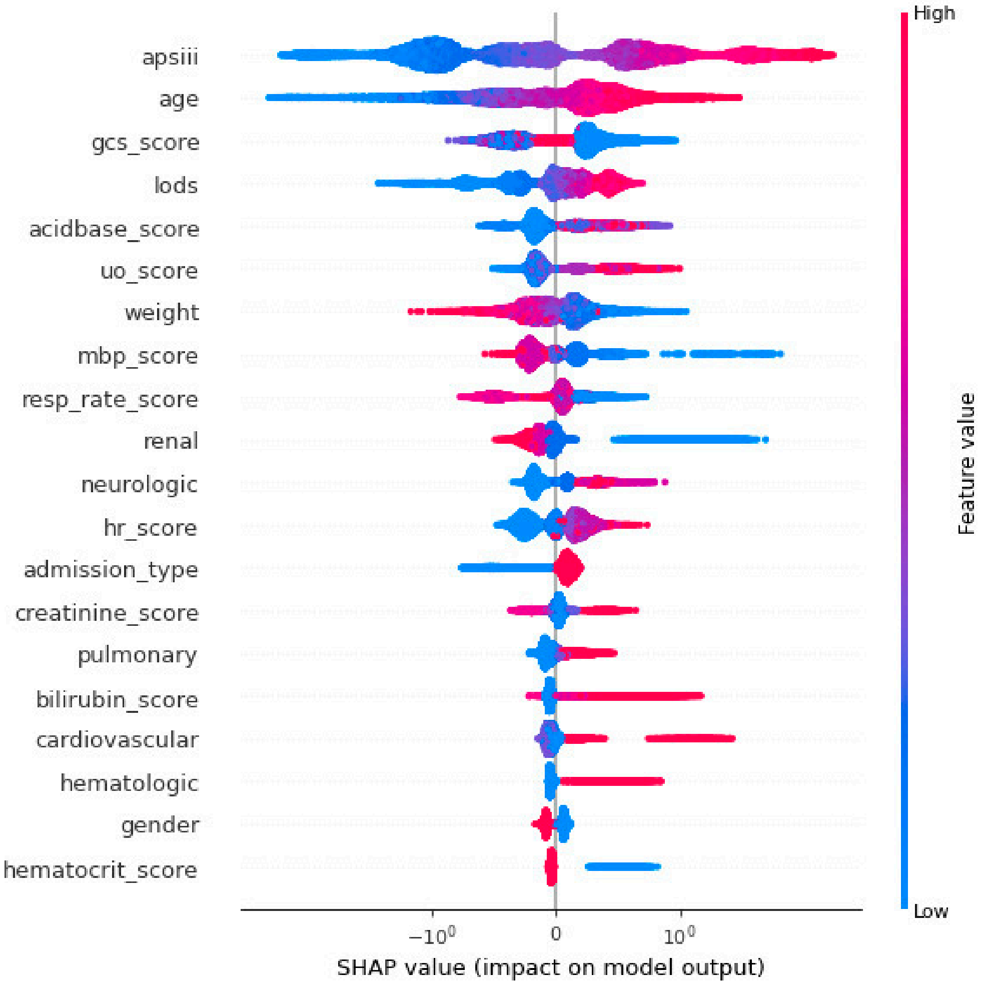

Supplementary Figure S2 SHAP values importance of all features in the Xgboost model

Supplement Table S1 Hyperparameter values of models

Models	Hyperparameters	Values
XGBoost	Learning rate	0.1
	Maximum tree depth	3
	Minimum sum of instance weight	1
	The subsample ratio of columns	0.8
	Subsample	0.8
	Number of trees	380
SVM	Kernel	linear
	Penalty parameter of the error term	9
Logistic Regression	Solver	liblinear
	Penalty	L2
Decision Tree	Maximum tree depth	5
	The minimum samples required to split an internal node	2
	The minimum samples required to be at a leaf node	10
